# Supplementary figures and images for: Environmental and Lifestyle Cancer Risk Factors: Shaping Extracellular Vesicle OncomiRs and Paving the Path to Cancer Development
Source: Cancers (Basel). 2023 Aug 29;15(17):4317. doi: 10.3390/cancers15174317 (PMC10486808; doi:10.3390/cancers15174317)

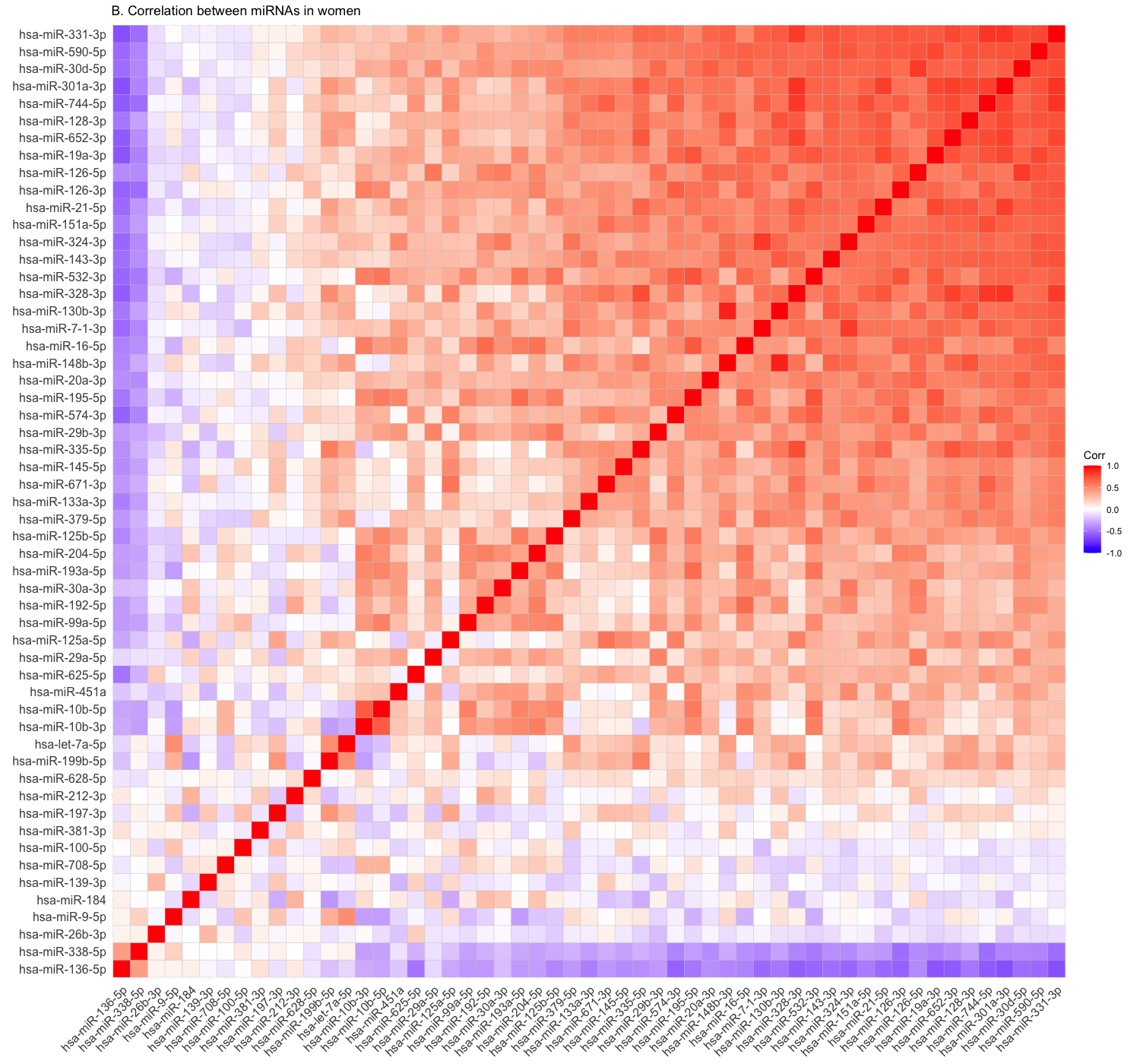

Supplement: Supplementary file 1 [file cancers-15-04317-s001.zip › Supplementary Figure 1A.png]

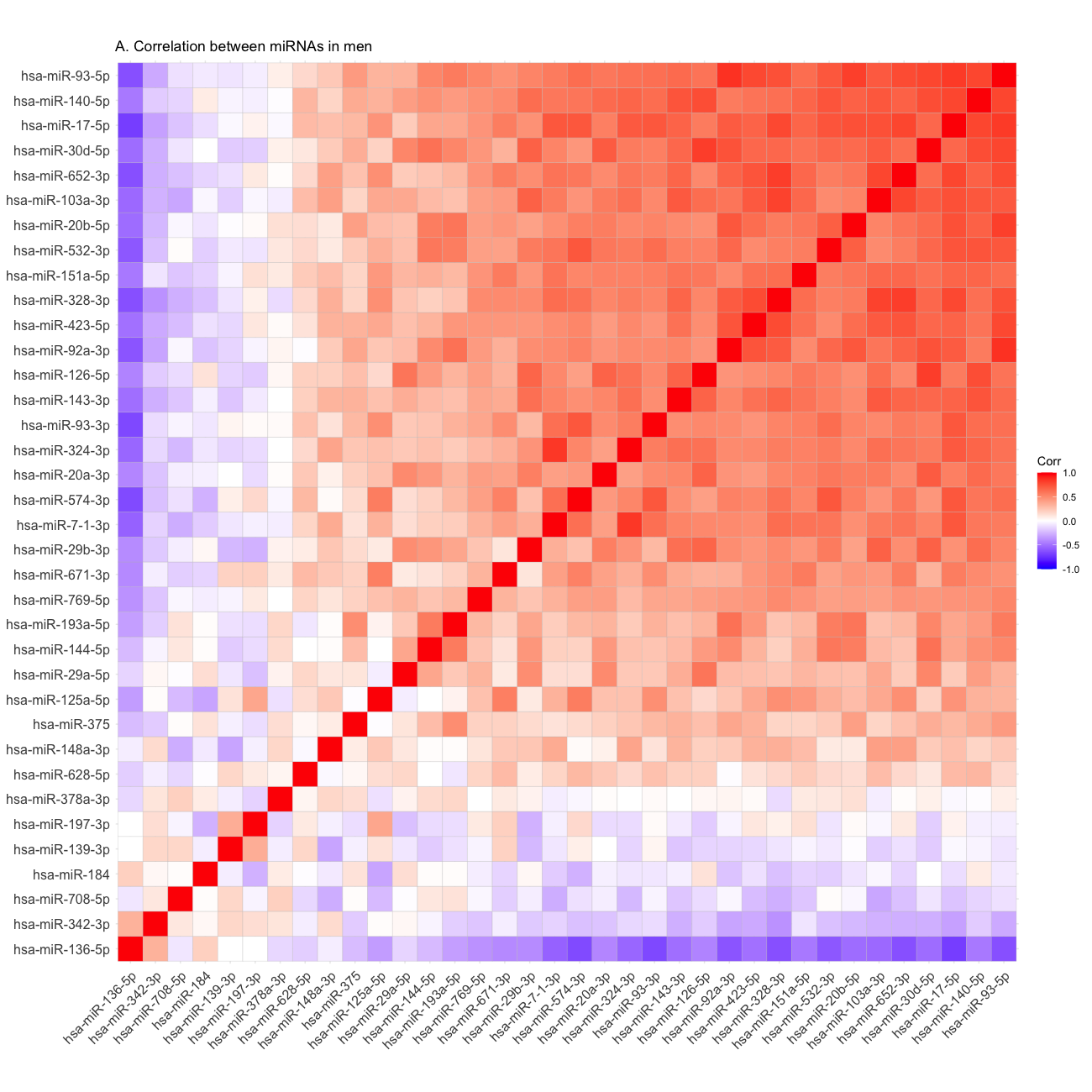

Supplement: Supplementary file 1 [file cancers-15-04317-s001.zip › Supplementary Figure 1B.png]
